# Molecular Neuroimaging of the Dopamine Transporter as a Patient Enrichment Biomarker for Clinical Trials for Early Parkinson's Disease

**DOI:** 10.1111/cts.12619

**Published:** 2019-03-18

**Authors:** Klaus Romero, Daniela Conrado, Jackson Burton, Timothy Nicholas, Vikram Sinha, Sreeraj Macha, Malidi Ahamadi, Jesse Cedarbaum, John Seibyl, Kenneth Marek, Peter Basseches, Derek Hill, Ed Somer, Jill Gallagher, David T. Dexter, Arthur Roach, Diane Stephenson, for the Critical Path for Parkinson's (CPP) Consortium, the Parkinson's Progression Markers Initiative (PPMI)

**Affiliations:** ^1^ Critical Path Institute Tucson Arizona USA; ^2^ Pfizer Groton Connecticut USA; ^3^ Merck Whitehouse Station New Jersey USA; ^4^ Biogen Cambridge Massachusetts USA; ^5^ Molecular Neuroimaging New Haven Connecticut USA; ^6^ Panoramic Digital Health Saint Pierre de Chartreuse France; ^7^ General Electric Little Chalfont UK; ^8^ Parkinson's UK London UK

## Abstract

The Critical Path for Parkinson's (CPP) Imaging Biomarker and Modeling and Simulation working groups aimed to achieve qualification opinion by the European Medicines Agency (EMA) Committee for Medical Products for Human Use (CHMP) for the use of baseline dopamine transporter neuroimaging for patient selection in early Parkinson's disease clinical trials. This paper describes the regulatory science strategy to achieve this goal. CPP is an international consortium of three Parkinson's charities and nine pharmaceutical partners, coordinated by the Critical Path Institute.

**Study Highlights**

**WHAT IS THE CURRENT KNOWLEDGE ON THE TOPIC?**

✓ dopamine transporter (DAT) neuroimaging has been used as a diagnostic aid for Parkinson’s disease. The regulatory strategy toward DAT qualification as an enrichment biomarker is described in this paper.

**WHAT QUESTION DID THIS STUDY ADDRESS?**

✓ This effort formally quantified the utility of DAT as a clinical trial enrichment tool, through the development of a quantitative disease progression model.

**WHAT DOES THIS STUDY ADD TO OUR KNOWLEDGE?**

✓ The underlying disease progression model allows sponsors to define trial‐specific enrichment strategies for DAT.

**HOW MIGHT THIS CHANGE CLINICAL PHARMACOLOGY OR TRANSLATIONAL SCIENCE?**

✓ The qualification of DAT as an enrichment biomarker is yet another example of how disease progression models are needed in order to qualify clinical biomarkers.

There is an urgent need for novel treatments against Parkinson's disease (PD), especially intended to target the earlier stages of disease progression. Clinical trials to evaluate these drug candidates require tools that allow an optimal selection of trial participants, for instance, enrollment of homogeneous populations in terms of their expected disease progression.^1^

The US Food and Drug Administration has approved molecular dopamine transport (DAT) neuroimaging as an adjunct diagnostic evaluation to help differentiate essential tremor from tremor due to parkinsonian syndromes, whereas the European Medicines Agency (EMA) approved DAT imaging to detect loss of functional dopaminergic neuron terminals in the striatum.^2^, ^3^ The role of DAT imaging as a drug‐development tool (DDT) to optimize predictions of motor progression was recognized as having potential as an enrichment biomarker for clinical trials. The EMA Qualification of Novel Methodologies in Drug Development pathway is a specific regulatory mechanism that facilitates qualification of DDTs, such as this application of DAT imaging.^4^

This paper describes the strategy to achieve qualification of this enrichment biomarker through this EMA pathway. This strategy was designed and executed by the Critical Path for Parkinson's (CPP), a public–private partnership coordinated by the Critical Path Institute and funded by Parkinson's UK and its industrial partners.^5^ The CPP brings together subject matter experts representing the pharmaceutical industry, academia, and regulatory authorities. The CPP working groups developed and executed a comprehensive qualification analysis plan, following the EMA guidance document for the Qualification of Novel Methodologies in Drug Development. The results from the modeling analyses were submitted to the EMA, which led to a qualification opinion for DAT imaging as an enrichment biomarker for clinical trials in early motor PD.^6^ The findings were presented via Scientific Advice mechanism with the EMA's Scientific Advice Working (SAWP) Party on July 4, 2017, leading to the Qualification Opinion mechanism with EMA's Committee for Medicinal Products for Human Use (CHMP), on May 28, 2018.^7^

## Methods

### Regulatory pathway with the EMA

The regulatory strategy was designed around the pathway for the qualification of novel methodologies in drug development.^4^ This process started with the submission of a letter of intent and briefing package that included: (i) the proposed context of use (COU; **Table** 1), (ii) a comprehensive analysis plan (e.g., statistical model development and evaluation, assessment of magnitude of motor scores worsening, and enrichment utility), and (iii) target data sets to support the proposed analysis plan. This, in turn, triggered a formal review by the EMA's SAWP, followed by a face‐to‐face meeting in which the SAWP issued formal scientific advice for optimization and finalization of the COU statement and analysis plan. Subsequently, the modeling analyses were executed, and, upon their completion, a final qualification package was submitted to the EMA for a final review and SAWP meeting. This final meeting was aimed at reaching a final determination if the presented results constituted supporting evidence for the proposed COU. Afterward, the SAWP met with the CHMP, who made the final decision to issue a qualification opinion.^8^

**Table 1:** Description of context of use statement

COU component	Description
General area	Enrichment biomarker for clinical trials in early motor PD
Target population for use	Patients with early motor PD, defined by the UK Brain Bank Criteria^10^ as outlined below: Having at least two of the following: resting tremor, bradykinesia, rigidity (must have either resting tremor or bradykinesia); OR either asymmetric resting tremor or asymmetric bradykinesia. o Based on above criteria, combinations could include: resting tremor/bradykinesia, bradykinesia/rigidity, and resting tremor/rigidity. o Symptom(s) or signs may include bradykinesia, a 4−6 Hz resting tremor, muscle rigidity, or postural instability not caused by primary visual, vestibular, cerebellar or proprioceptive dysfunction. Hoehn and Yahr stage I or II at baseline. o Although postural instability is a common feature in PD, based on the inclusion criterion of Hoehn and Yahr Stage I or II, postural instability would not be expected in the target population.
Stage of drug development for use	All clinical stages of early PD drug development, including proof‐of‐concept, dose‐ranging through to confirmatory clinical trials. This is not intended for candidate therapies for more advanced stages of PD, such as drugs to treat L‐Dopa–induced dyskinesia.
Intended application	Purpose: The objective of this project is to apply DAT imaging as a biomarker tool to enrich subjects for clinical trials in early symptomatic PD by identifying subjects with a DAT deficit for possible inclusion into the study and excluding subjects who are unlikely to progress due to the lack of dopamine deficiency in the brain. The DAT imaging is intended to be used after the clinical criteria for early PD have been satisfied. Potential candidates for PD clinical trials will be evaluated for the presence of at least two motor signs of PD, as described in the target population description of this section (according to the PPMI and PRECEPT criteria). Those individuals are then evaluated according to the UK Brain Bank step 1 Criteria for PD. If the two conditions above are met, subjects will undergo the trial‐specific inclusion/exclusion criteria and further clinical assessment for atypical Parkinsonian syndromes. As a final step in the subject‐selection process, molecular imaging of DAT will be performed to detect the presence or absence of DAT‐deficiency and identify and exclude subjects defined as SWEDDs. Such baseline categorization of DAT‐deficiency can be applied as an enrichment biomarker that, in combination with specific clinical signs, can more accurately predict disease progression of motor disability in early PD patients. Such progression will be expressed by the motor scores of the UPDRS or MDS‐UPDRS scales, which constitute reliable outcomes of disease progression in PD. Baseline categorization of DAT‐deficiency can be applied as a subject selection biomarker to enrich trial populations with patients more likely to progress in the motor scores of UPDRS or MDS‐UPDRS scale (parts II and III) over the course of clinical trials, which may be up to 2 years in duration. The purpose is to exclude patients who are unlikely to show disease progression (SWEDD), and consequently to increase the probability of the trial conclusively demonstrating the effect of an effective drug in clinical trials for therapeutic interventions for early PD. Those individuals who are not SWEDDs and who meet all the other selection criteria will be enrolled into the trial and randomized as per the specified study design. The use of DAT imaging would allow the exclusion of subjects unlikely to have the diagnosis of PD and, therefore, prevent them from unjustified exposure to experimental PD‐specific therapies with inherent safety and tolerability risks without anticipated benefit. The application is relevant to both symptomatic and disease‐modifying candidate therapies for early PD and is independent of the mechanism of action of the new drug. The use of DAT imaging for diagnostic applications are out‐of‐scope for this proposed COU.
Critical parameters for the context‐of‐use	The context‐of‐use specifies that reductions of DAT, as assessed by SPECT neuroimaging, will be utilized as an adjunct to clinical assessments for the purposes of enriching the patient population with subjects who have increased likelihood of having idiopathic PD. The subjects will have an objectively confirmed motor impairment with alternative identifiable causes of motor impairment appropriately excluded through clinical means prior to the use of DAT neuroimaging. SPECT neuroimaging procedures and methodologic aspects of imaging will be performed qualitatively in accord with the tracer manufacturer's specifications and consistent with the methods currently used in the multisite PPMI study. The proposed analysis of DAT SPECT images is by visual assessment by trained blinded readers and analysis is to be carried out by a single site. Such processes are expected to generate sufficiently accurate, reproducible, and robust assessment of DAT neuroimaging to facilitate clinical trial enrichment.

COU, Context of Use; DAT, dopamine transporter; MDS‐UPDRS, Movement Disorder Society‐Unified Parkinson’s Disease Rating Scale; PD, Parkinson's disease; PPMI, Parkinson Progression Markers Initiative; PRECEPT, Parkinson Research Examination of CEP‐1347 Trial; SPECT, single photon emission computed tomography; SWEDDs, scans without evidence of dopaminergic deficits; UPDRS, Unified Parkinson's Disease Rating Scale.

### Data sources

**Figure 1:**
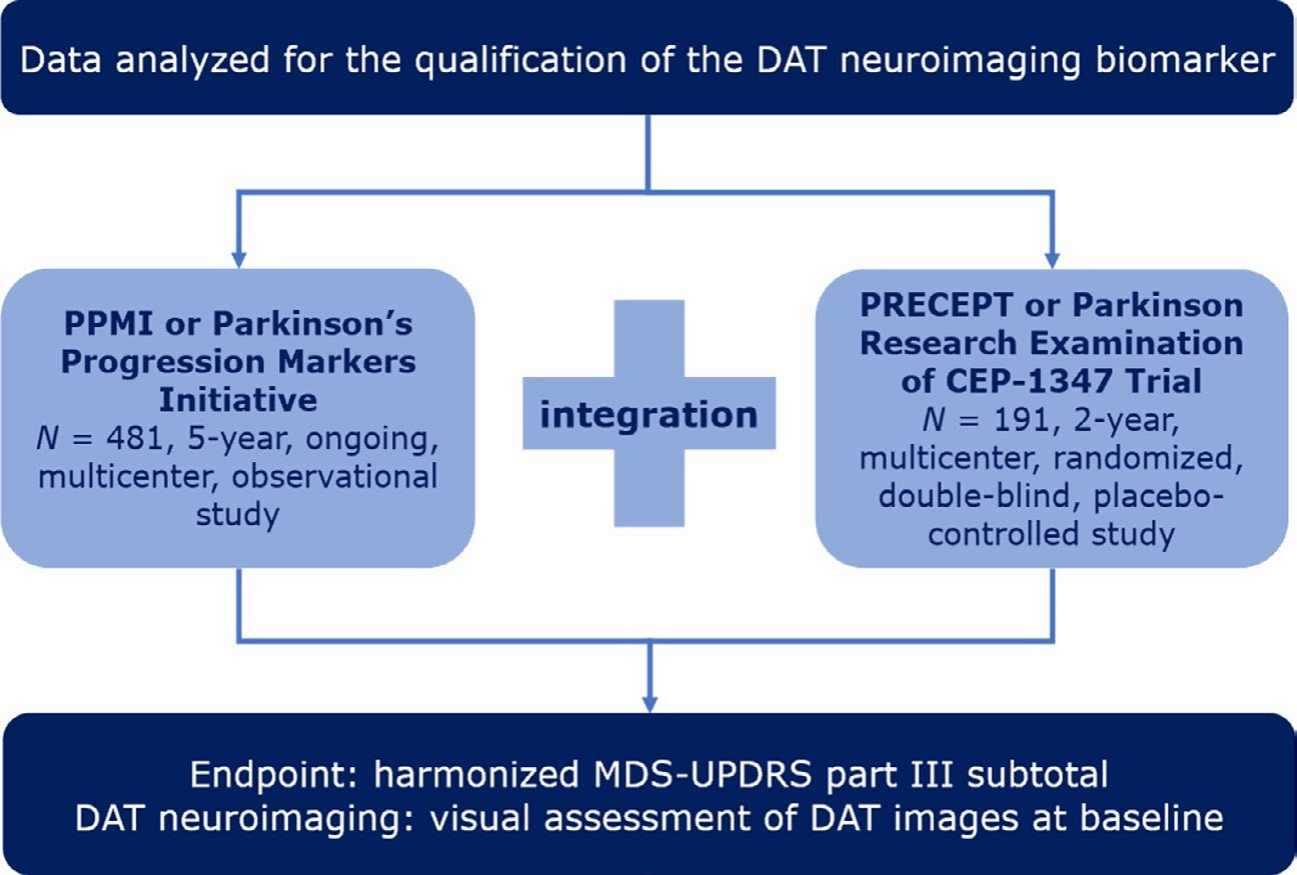
Integration of data sources and harmonization of motor scores. DAT, dopamine transporter; MDS‐UPDRS, Movement Disorder Society‐Sponsored Revision of the Unified Parkinson Disease Rating Scale; PRECEPT, Parkinson Research Examination of CEP‐1347 Trial.

Integrated patient‐level data from the Parkinson's Progression Markers Initiative (PPMI) and the Parkinson Research Examination of CEP‐1347 Trial (PRECEPT) were analyzed.^5^, ^6^ These data correspond to a total of 672 subjects diagnosed with early‐stage PD and a total of 4,521 observations in the baseline to 25‐month interval^7^ (**Figure** 1).

### Definition of biomarker status

Dopamine transporter neuroimaging status was treated as a binary covariate based on visual reads, with individuals classified as either having scans without evidence of dopaminergic deficit (SWEDD; or biomarker‐negative) or having scans with evidence of dopaminergic deficit (biomarker‐positive).

### Harmonization of motor scores

The transformation of the individual Unified Parkinson's Disease Rating Scale (UPDRS) part III subtotal score in PRECEPT to the respective Movement Disorder Society (MDS)‐UPDRS part III subtotal score in PPMI relied on a previously derived method.^9^ This allowed motor score integration across both studies.

### Statistical modeling and clinical trial simulation

As described by Conrado *et al*.,^7^ an early motor PD progression model was developed using the harmonized MDS‐UPDRS part III as the end point. Through this model, the difference in progression rate between SWEDDs and DAT deficit subjects and utility of DAT‐based enrichment was determined.

In the PD progression model, the time course of the harmonized motor scores was described using a linear mixed‐effects model. Prespecified covariates were the effect of biomarker status in baseline motor scores and progression rate and the effect of study in baseline motor scores to account for potential score differences between the PPMI and PRECEPT populations. Additional exploratory covariates included the effect of age in baseline motor scores and in progression rate given the neurodegenerative nature of PD and the effect of study in progression rate to investigate potential rate differences between the PPMI and PRECEPT. The final model included all the prespecified covariates (Eq. S1, **Supplementary Material**), and all statistically significant exploratory covariates.

Monte Carlo–based clinical trial simulations were performed to compare the statistical power vs. sample size in trials with and without DAT imaging enrichment. Enriched trials had only subjects with DAT deficit, whereas nonenriched trials included 15% of SWEDD subjects.^7^ The statistical power, defined herein as the probability of detecting a drug effect of 50% reduction in progression rate, was calculated as the proportion of trials for which the drug effect on progression rate was beneficial with a two‐tailed *P* value lower than 0.05.

Additional detailed information on the methods has been published in ref. 7.

## Results

### Disease progression model

**Figure 2:**
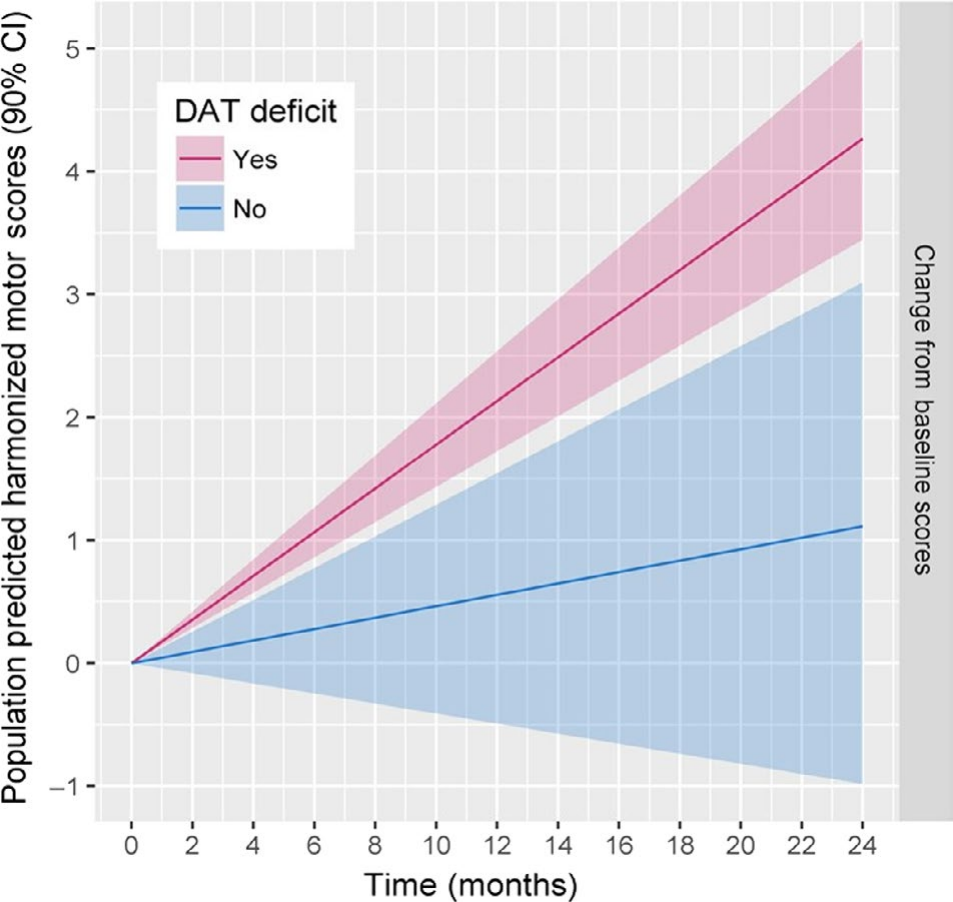
Predicted harmonized motor scores. Subjects with and without DAT, dopamine transporter (DAT) deficit have an average monthly progression in scores of 0.18 (90% confidence interval (CI): 0.14, 0.21) and 0.05 (90% CI: −0.04, 0.13) points/month, respectively (Image reproduced from ref. 7 https://doi.org/10.1111/cts.12492, is licensed under CC BY 4.0. ©2017 The authors.).

The final linear mixed‐effects model included: (i) effect of biomarker status on baseline, (ii) effect of biomarker status on progression rate, (iii) effect of study on baseline, and (iv) effect of age on baseline^7^ (**Figure** 2). Model diagnostics suggested an adequate fit of the longitudinal changes in the harmonized score.^7^ The main findings were:

- The estimated effect of SWEDD on progression rate was −0.13 points/month (90% confidence interval (CI): −0.23 to −0.04; one‐tailed *P* value = 0.01). This means that SWEDDs have an average monthly progression in the harmonized motor scores that is 0.05 (90% CI: −0.04 to 0.13) points/month or 0.13 point/month lower than those with DAT deficit (0.18 points/month; 90% CI: 0.14−0.21).
- The estimated effect of SWEDD on baseline was −7.69 (90% CI: −9.4 to −6.04) points; hence, SWEDDs have an average baseline harmonized motor score that is 7.69 points lower than those with DAT deficit.
- The estimated effect of year of age on baseline was 0.19 (90% CI: 0.14−0.24) points, which means that on average, the baseline harmonized motor score increases by 0.19 points for each year of age. Thus, the baseline score for a typical 60 year old subject with DAT deficit is expected to be 21.54 points.^7^

### Magnitude of motor scores worsening between biomarker statuses

The magnitude of motor scores worsening (defined as change from baseline at 24 months) in DAT deficit and SWEDD subjects was 4.28 (90% CI: 3.45−5.08) and 1.12 (90% CI: −0.98 to 3.1), respectively. The average difference between biomarker statuses was −3.16 (90% CI: −0.96 to −5.42) points, indicating that subjects with DAT deficit have an average of 3.16 points higher (worse) 24‐month change from baseline motor score than SWEDDs.

### Clinical trial simulations and statistical power

Simulated trial designs were placebo‐controlled, parallel, and crossover with total duration of 12 and 24 months. For each design, 2,000 enriched and nonenriched clinical trials were simulated, yielding a total of 8 scenarios. For such scenarios, DAT imaging‐based enrichment strategy was estimated to allow a 20–30% reduction of trial size.

## Discussion

The following were considered key issues to support the regulatory discussion:

1. Biomarker deficit status (SWEDD vs. DAT deficit) as a predictor of disease progression, even when the difference in baseline severity has been accounted for.

**Figure 3:**
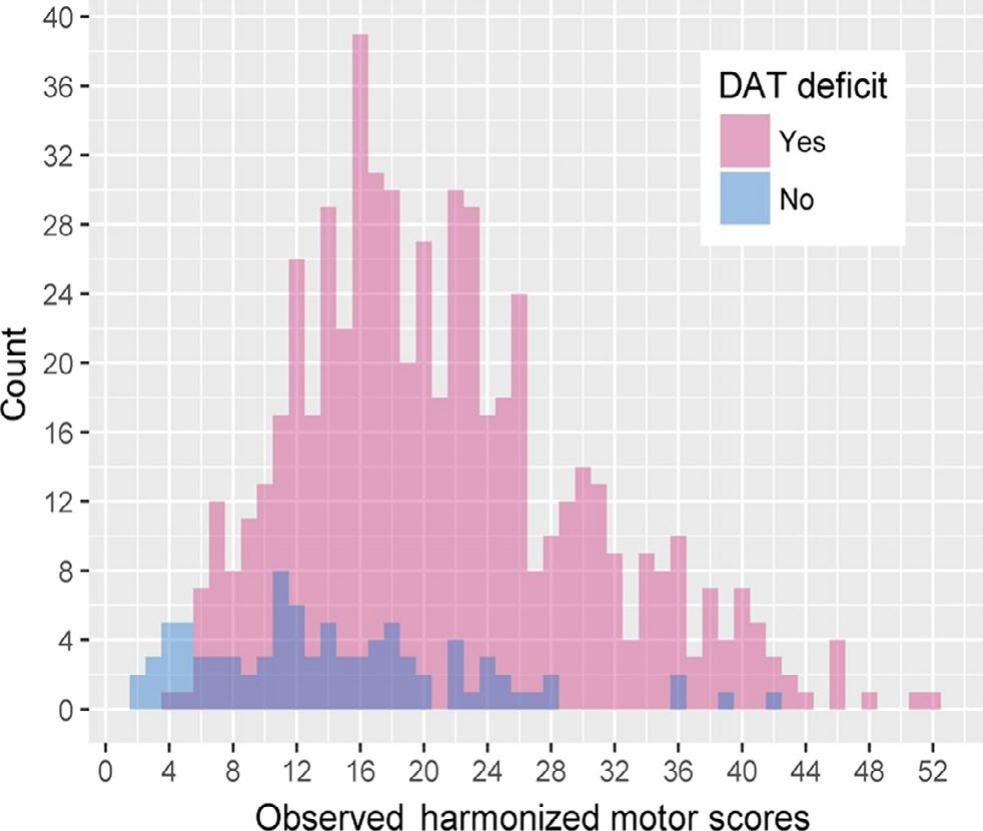
Distribution of baseline scores for SWEDD and dopamine transporter dopamine transporter (DAT) deficit subjects in the Parkinson Progression Markers Initiative and scans without evidence of dopaminergic deficit (PRECEPT) integrated data set. A baseline‐matched data set was produced to investigate the effect of baseline on rate of progression. PRECEPT, Parkinson Research Examination of CEP‐1347 Trial; SWEDD, scans without evidence of dopaminergic deficit.

Because the distribution of observed baseline motor scores shows some degree of overlap in the baseline scores between SWEDD and DAT deficit subjects (**Figure** 3), a baseline‐matched subset of the data was created. In this baseline‐matched subset, DAT deficit subjects were included only if there was more than one SWEDD subject with the same observed baseline score (rounded to zero decimal places); likewise, SWEDD subjects were included only if there was more than one DAT deficit subject with the same observed baseline score (rounded to zero decimal places). A supplementary statistical analysis was then performed using this baseline‐matched subset. Given the association between biomarker status and baseline motor scores, a baseline‐matched data set decreases the likelihood of confounding effects and helps investigate the separate contribution of baseline and biomarker status on the rate of progression. Results showed a significant difference in progression rate between the SWEDD and DAT deficit groups of −0.19 points/month (two‐tailed *P* value < 0.05) even after accounting for disease severity at baseline (**Table** 2).

**Table 2:** Parameter estimates from the supplementary analysis using the baseline‐matched subset (*N* = 463)

Parameter	Estimate	*P* value
Intercept or baseline score (points)	12.55	a
Effect of PRECEPT on baseline	0.59	NS
Effect of year of age on baseline	0.08	a
Effect of SWEDD on baseline	−2.41	a
Slope or progression rate (point/month)	−0.19	a
Effect of SWEDD on progression rate	−0.19	a
Effect of baseline on progression rate	0.03	a

NS, indicates two‐tailed *P* value > 0.05; PRECEPT, Parkinson Research Examination of CEP‐1347 Trial; SWEDDs, scans without evidence of dopaminergic deficits.

The estimated effect of SWEDD on progression rate of −0.19 points/month remains statistically significant even after accounting for the effect of baseline. SWEDDs have a progression rate that is 0.19 points/month lower than that observed for dopamine transporter deficit subjects.

a Indicates two‐tailed *P* value < 0.05.

A second supplementary analysis was performed on totality of data (*N* = 672), including effect of baseline disease severity on progression rate. Such analysis yielded a difference in progression rate between SWEDD and DAT deficit subjects of −0.24 points/month (*P* value < 0.05). This provides further evidence for DAT imaging as an actual predictor of disease progression (**Table** 3).

**Table 3:** Parameter estimates from the supplementary analysis using the entire data set (*N* = 672)

Parameter	Estimate	*P* value
Intercept at baseline scores (points)	12.71	a
Effect of PRECEPT on baseline	0.79	NS
Effect of year of age on baseline	0.15	a
Effect of SWEDD on baseline	−7.70	a
Slope or progression rate (point/month)	−0.31	a
Effect of SWEDD on progression rate	−0.24	a
Effect of baseline on progression rate	0.02	a
Additional effect of baseline on progression rate in SWEDD	0.02	a

DAT, dopamine transporter; NS, indicates two‐tailed *P* value > 0.05; PRECEPT, Parkinson Research Examination of CEP‐1347 Trial; SWEDD, scans without evidence of dopaminergic deficit.

Further support for the DAT imaging status as a predictor of disease progression rate.

a Indicates two‐tailed *P* value < 0.05.

1. Similarities between SWEDD and DAT deficit subject entry criteria in PPMI and PRECEPT.

The clinical enrollment criteria for SWEDD and DAT deficit subjects were equivalent in PPMI and PRECEPT (i.e., SWEDD subjects were not recruited as a separate cohort and were not identified until after recruitment). In PPMI, DAT imaging was performed after subjects met the clinical criteria (as described in **Table** 1). SWEDD subjects were asked to remain enrolled and then longitudinally followed for 2 years after consenting to remain in the study. The baseline characteristics of PPMI and PRECEPT are similar (**Table** 4) and representative of future clinical trial populations with early motor PD.

**Table 4:** Baseline characteristics of SWEDD and DAT deficit subjects across PPMI and PRECEPT

Baseline	PPMI		PRECEPT	
DAT imaging status	DAT Deficit	SWEDD	DAT Deficit	SWEDD
Sample size	418	63	165	26
Sex, %	Female (35), Male (65)	Female (38), Male (62)	Female (33), Male (67)	Female (38), Male (62)
Age in year, mean (range)	61 (33−84)	60 (38−78)	59 (31−82)	60 (32−84)
Harmonized motor scores, mean (range)	21 (4−51)	14 (2−42)	22 (7.1−52)	14 (5.3−28)

DAT, dopamine transporter; PPMI, Parkinson Progression Markers Initiative; PRECEPT, Parkinson Research Examination of CEP‐1347 Trial; SWEDD, scans without evidence of dopaminergic deficit.

1. Representativeness of PPMI and PRECEPT of the external SWEDD and DAT deficit population.

Subjects enrolled in PPMI and PRECEPT are representative of the population likely to participate in PD clinical trials and the target population to be selected by DAT neuroimaging.

Clinical studies will continue to evaluate treatment response in earlier stages of PD, where it is known that there is greater uncertainty in selecting participants based on clinical criteria alone. Enrolling more homogeneous populations in these studies can help optimize clinical trial design and avoid exposing subjects who are less likely to progress to unknown test drugs.

1. Similarity between SWEDD and DAT deficit subject imaging acquisition in PPMI and PRECEPT.

The technical aspects of the data acquisition of DAT single photon emission computed tomography (SPECT) images were identical between SWEDD and DAT deficit subjects and not a reason for dopaminergic differences between SWEDD and PD subjects. For PPMI, all subjects (SWEDD and DAT deficit) were aligned in terms of their imaging acquisition protocol, including the time interval between injection and SPECT reading (4 hours in duration). For PRECEPT, all imaging was done on a single research SPECT camera, and the data were managed by the core laboratory research group. CPP concluded that the dopaminergic differences between SWEDD and DAT deficit subjects are not due to variations in image acquisition. The potential impact of medications on DAT imaging was discussed. Symptomatic agents have been shown not to impact ligand binding. Drugs that bind to DAT (cocaine and amphetamines) were not permitted for PPMI enrollment. Antidepressants have been investigated, with no impact on the outcome of the visual assessments for DAT deficiency.

1. SWEDD subjects who experienced progression.

Possible reasons that could explain why some SWEDD subjects in the data set experienced progression: (i) they did not progress as typical patients with PD and were possibly dystonic tremor subjects, (ii) the test performed in these subjects was affected by external conditions that might have led to less signal in the striatum as compared with the occipital cortex and, hence, a false negative.

As per the COU statement, diagnostic applications for DAT imaging are out of the scope of this work. DAT imaging could identify subjects with a homogeneous motor decline, allowing trial enrichment and meaningful reduction of sample size, regardless of the ultimate diagnosis. Moreover, as aforementioned, DAT imaging aspects were identical between SWEDD and DAT deficit subjects.

1. Consideration of sensitivity, specificity, and predictive values for DAT imaging in the proposed COU.

As discussed, DAT imaging status is a statistically and clinically significant predictor of disease progression. Clinical trial simulations based on the underlying model allow the estimation of trial‐specific DAT imaging‐based enrichment magnitudes regardless of the ultimate diagnosis, thus providing a useful drug development tool to optimize decision making. As such, traditional concepts such as sensitivity, specificity, and predictive values, commonly applied to diagnostic biomarkers, are not of relevance in this enrichment context.

## Conclusions

These findings show that a DAT‐SPECT finding of integrity of presynaptic dopaminergic terminals in a case of suspected PD is associated with a good prognosis, whatever the ultimate diagnosis. Exclusion of SWEDD subjects from future clinical trials will improve the chance of determining clinical benefit of new drug candidates to treat PD.

The EMA pathway for the Qualification of Novel Methodologies in drug development provides a valuable mechanism for the review and regulatory endorsement of DDTs, such as biomarkers and quantitative drug development tools. The final qualification opinion for DAT‐SPECT imaging is publicly available.^8^ Regulatory endorsement provides sponsors with the necessary confidence to apply novel approaches to optimize drug development, which is much needed for neurodegenerative conditions such as PD.

Model‐informed biomarker qualification is an efficient method to evaluate the utility of biomarker candidates for specific COU statements. The modeling approach presented herein relied on the time course of motor scores worsening. Traditional concepts applied to diagnostic biomarkers (e.g., sensitivity, specificity, and predictive values) were not needed to demonstrate the utility of DAT imaging as an enrichment biomarker for trials in early motor PD.

## Funding

This work was funded by the CPP Consortium, a public–private partnership mainly funded by Parkinson's UK.

## Conflict of Interest

T.N. is an employee of Pfizer. V.S., S.M., M.A. and P.B. are employees of Merck. J.C. is an employee of Biogen. J.S. and K.M. and employees of Molecular Neuroimaging. D.H. is a former employee of IXICO. E.S. is an employee of GE Healthcare.

## Author Contributions

K.R., D.C., J.B., T.N., V.S., S.M., M.A., J.C., J.S., K.M., P.B., D.H., E.S., J.G., D.T.D., A.R., and D.S. wrote the manuscript. K.R., D.C., J.B., T.N., V.S., S.M., and M.A. designed the research. D.C. performed the research.
